# Does Fruit and Vegetable Consumption During Adolescence Predict Adult Depression? A Longitudinal Study of US Adolescents

**DOI:** 10.3389/fpsyt.2018.00581

**Published:** 2018-11-13

**Authors:** Erin Hoare, Meghan Hockey, Anu Ruusunen, Felice N. Jacka

**Affiliations:** ^1^Food & Mood Centre, IMPACT SRC, School of Medicine, Deakin University, Geelong, VIC, Australia; ^2^Department of Psychiatry, Kuopio University Hospital, Kuopio, Finland; ^3^Institute of Public Health and Clinical Nutrition, University of Eastern Finland, Kuopio, Finland

**Keywords:** depression, adolescents, fruit, vegetables, adulthood

## Abstract

The relationship between better diet quality and decreased depression across the life span is consistent and compelling. Fruit and vegetable consumption has been of particular interest. The nutritional benefits from the consumption of fruits and vegetables may mitigate non-communicable diseases and promote brain and mental health. This study aimed to determine whether fruit and vegetable consumption during adolescence was associated with a reduced risk of developing depression in adulthood in a large, representative sample of US individuals. Data from the Add Health Study were analyzed, which included 3,696 participants who were aged approximately 17 years at baseline (1994–1995), and 29 years at follow-up (2007–2008). The Center for Epidemiologic Studies Depression Scale was used to assess depression and a self-report item asked how many times the participant consumed fruit/vegetables on the previous day. Individuals who were depressed at both times points had the highest proportion who failed to consume any fruit (31%) or vegetables (42%) on the previous day. Fruit and vegetable consumption did not predict of adult depression in fully adjusted models. Cross sectional associations existed for diet and adolescent depression only. Our initial findings supported fruit and vegetable consumption as being protective against adult depression, but this association was subsequently attenuated on adjustment for other relevant factors. Future research will benefit from more precise measures of dietary intakes.

## Introduction

Healthful diets for the prevention and treatment of depression is a current major focus in nutritional psychiatric research. In particular, the Mediterranean Style Diet, traditional wholefood diets, and anti-oxidant rich foods have been promoted as supportive of positive mental health (1–4). Depression is a highly complex mental disorder and is the leading contributor to the global burden of disease (5). The causes and risk factors of depression span individual, familial, environmental, societal and other domains, thus prevention strategies are needed that account for the complexity within which depression occurs (6). Individuals experiencing depression are at greater risk of experiencing comorbid lifestyle driven diseases such as overweight/obesity, cardiovascular disease and some cancers (7). These conditions are all associated with underlying poor diet and it is therefore highly plausible that prevention strategies that target healthier diet could hold dual physical and mental health benefits. It is recognized that experiencing depression and associated symptoms such as low motivation, can also lead to poor dietary choices and overall unhealthy eating habits particularly as a form of coping (8). Indeed the typical diet of individuals living with mental illness is poorer at the population level, compared to individuals without mental illness (9). The diet and depression relationship is therefore assumed to be bi-directional. Whilst the etiology of depression is highly complex, targeting diet for prevention holds great promise given the population reach and the known dual benefits for physical health and non-communicable disease prevention.

The underlying components of diets considered beneficial for mental health are all similar in that they all promote the regular consumption of fruits and vegetables (4). Fruits and vegetables are micronutrient and anti-oxidant rich, which is thought to be protective against oxidative stress in the body (10, 11). In addition, vitamins and minerals found in fruit and vegetables have been shown to support healthy emotional and cognitive functioning (12). Diets high in fruit and vegetables have also been shown to impact directly on brain health. A study published in 2015 found that healthier dietary patterns, high in fruit and vegetables, were associated with larger hippocampal volume over time, compared to Western-style, unhealthy diets, low in fruit, and vegetables (13).

Evidence suggests that both low rates of common mental disorders including depression, low mood, stress, and anxiety (14, 15), and higher rates of positive mental health including life satisfaction, positive mood, and perceived life flourishing (16), co-occur with fruit and vegetable intake. Furthermore, research suggests that a dose-response relationship exists, whereby each serve increase in fruit and vegetables is associated with an improvement in mental health (17, 18). Given the evidence to date, treatment trials have been conducted examining the impact of dietary changes, including increased fruit and vegetable consumption, on depressive symptoms with significant positive outcomes being reported (3, 19).

Despite evidence to date, there are some research gaps in terms of the relationship between fruit and vegetable intake for the prevention of depression at a population-level. In particular, while it is known that a life span approach should be adopted, it remains unknown what the unique preventive potential of fruit and vegetable consumption during adolescence might be for reducing the risk of depression later in adulthood. This may be particularly critical given that depression often first occurs during adolescence (20). In addition, dietary habits formed in adolescence often track into adulthood, which has important implications for physical health later in life (21). Investigating the predictive potential of fruit and vegetable consumption during adolescence and adult depression outcomes thus has important implications for population-level prevention initiatives.

The aim of this study is to determine whether adolescent fruit and vegetable consumption predicts adult depression, and whether this predictive potential is independent of other factors known to be related to both diet and mental health outcomes, such as overweight/obesity, socio-economic status, and physical activity.

## Methods

### Study design

Add Health is a longitudinal, US population-based study of adolescent health (22). During the 1994–1995 school year, 132 secondary schools participated in a questionnaire data collection, which reflected a nationally representative sample of students aged between 12 and 18 years (*n* = 20,745). Additionally, four waves of in-home interviews were conducted in 1994–1994 (Wave 1), 1996 (Wave 2), 2001–2002 (Wave 3), and 2007-2008 (Wave 4), spanning a total of 18 years. The sampling strategy was stratified random sample that represented the entire US in regards to region, urban/rurality, size, and ethnicity. Further detailed information has been published elsewhere (22).

Our study examined data from the original participating adolescents from Wave 1, who also completed follow-up data collection at Wave 4, and had complete, public accessible data available (*n* = 4,807). Sample weights were employed to account for the baseline probabilities and potential clustering effects of school to which participant attended. Ethics approval for the Add Health study was approved by the Institutional Review Board of the University of North Carolina, Chapel Hill. Public-use data are available for research purposes and were accessed from http://www.cpc.unc.edu/projects/addhealth

### Depression

Depression was measured by the Center for Epidemiologic Studies Depression Scale (CES-D) (23) at baseline in Wave 1 (1994–1995, mean age 16 years) and at Wave 4 (2007–2008, mean age 29 years). The CES-D is often used in population level epidemiological studies to measure the presence of symptoms typically resembling depression. The questionnaire includes items relating to how often in the past week the individual experienced symptoms such as restless sleep, feelings of loneliness and trouble in concentrating. Responses range from 0 to 3, where 0 = rarely or never and 3 = most or all of the time. Higher scores reflect greater depressive symptoms. The CES-D shows good sensitivity, specificity and high internal consistency and has been previously used across a broad range of age groups including adolescents (24). Reliability and validity has been previously shown.

At Wave 1, the 20-item CES-D was administered, and at Wave 4, participants received the 10-item version. A score of 16 or higher on the CES-D-20 and 11 or higher on the CES-D-10 was used to define probable depression, as per recommendations (23, 25). Adult (Wave 4) depression was the main outcome of interest and adolescent depression was controlled for as a covariate in analyses. We also ran models to examine the cross-sectional relationship between adolescent depression and fruit and vegetable consumption.

### Fruit and vegetable consumption

Fruit and vegetable consumption was the main predictor of interest in this study. During Wave 1, participants completed questionnaires on habitual dietary intake including items asking whether they had eaten fruit or vegetables on the previous day. Specifically, participants were asked “How often did you eat fruit or drink fruit juice yesterday?” with responses “didn't eat,” “ate once” or “ate twice or more.” The same item with response options was asked for vegetable consumption. Single-item dietary measures for fruit and vegetable consumption are widely used in population level epidemiological questionnaires and have been shown to be indicative of overall dietary quality (26).

### Covariates

Demographic, health behavioral, and anthropometric variables were selected for inclusion, based on evidence demonstrating potential to confound the relationship between diet and depression. Wave 1 questionnaires asked information relating to participants age, sex, total household income during 1994 (reported by parents) and ethnicity. Health behaviors including smoking status and physical activity levels were assessed at Wave 1 asking participants to report the number of times during the past week they did exercise, such as jogging, walking, karate, jumping rope, gymnastics, or dancing. Smoking status was also self-reported. Height and weight were objectively measured at Wave 4 and converted into body mass index, which was further converted into healthy weight and overweight/obese weight status categories (27). Because objective measures were only taken at Wave 4 (and not Wave 1), these data were used to control for weight status in analyses.

### Statistical analyses

All analyses were conducted in Stata/SE 15.0 (Stata Corporation) and significance was assumed at *p* < 0.05. Total CES-D scores were calculated and dichotomised using the cut off ≥16 for Wave 1 (CES-D-20) and ≥11 for Wave 4 (CES-D-10) as recommended by previous research (28).

Logistic regression models were used to examine whether adult depression was significantly predicted by adolescent fruit and vegetable consumption. Odds ratios with 95% confidence intervals assessed these relationships, with outcomes being whether or not depression was present (0 = no depression, 1 = depression present). Models were sex-specific given the known unique experiences of depression specific to sex. Univariate relationships were examined (Model 1) and then baseline depressive status were added (Model 2). Covariates age, household income during adolescence, ethnicity, physical activity, smoking status, and weight status were adjusted for in Model 3.

## Results

Participants were approximately 16 years at adolescence (baseline) and 29 years at adulthood (follow-up) (Table 1). Most participants were of Caucasian decent and depression significantly varied by ethnicity. The mean household income during adolescence was significantly lower among those with adult depression compared to those without adult depression. Physical activity and smoking status during adolescence, and adult overweight/obesity did not differ between those depressed as adults and those not depressed.

**Table 1:** Participant characteristics, expressed as n (%) unless otherwise specified.

	**Not depressed in adulthood (*n* = 3,003)**	**Depressed in adulthood (*n* = 693)**	**Total (*n* = 3,696)**
**Age, mean (SD)**			
Baseline	15.9 (1.7)	15.9 (1.8)	15.9 (1.7)
Follow-up	28.9 (1.7)	28.9 (1.8)	28.9 (1.7)
**Ethnicity**			
Caucasian	2189 (72.9)	436 (62.9)	2625 (71.0)
African American	591 (19.7)	193 (27.9)^*^	784 (21.2)
Native American	34 (1.1)	Supressed	Supressed
Asian/pacific islander	85 (2.8)	Supressed	Supressed
Other	104 (3.5)	38 (5.5)	142 (3.8)
Household income during adolescence m SD (‘000s)	50.8 (57.0)	40.2 (45.6)^*^	48.8 (55.2)
Smokers	565 (18.7)	150 (21.7)	712 (19.3)
**Physical activity^a^**			
Not at all	508 (16.9)	108 (15.6)	616 (16.7)
1 or 2 times	942 (31.4)	212 (30.6)	1,154 (31.2)
3 or 4 times	761 (25.3)	177 (25.5)	938 (25.4)
5 or more times	792 (26.4)	196 (28.3)	988 (26.7)
Overweight/obese in adulthood	2,013 (67.0)	469 (67.7)	2,482 (67.2)
Depression during adolescence	491 (16.4)	289 (41.7)^*^	780 (21.1)

* *Significant (p < 0.05) difference between groups not depressed in adulthood and depressed in adulthood*.

*Abbreviations: Standard deviation, SD*.

a *Participants were asked the number of times during the past week they did exercise, such as jogging, walking, karate, jumping rope, gymnastics, or dancing*.

**Figure 1:**
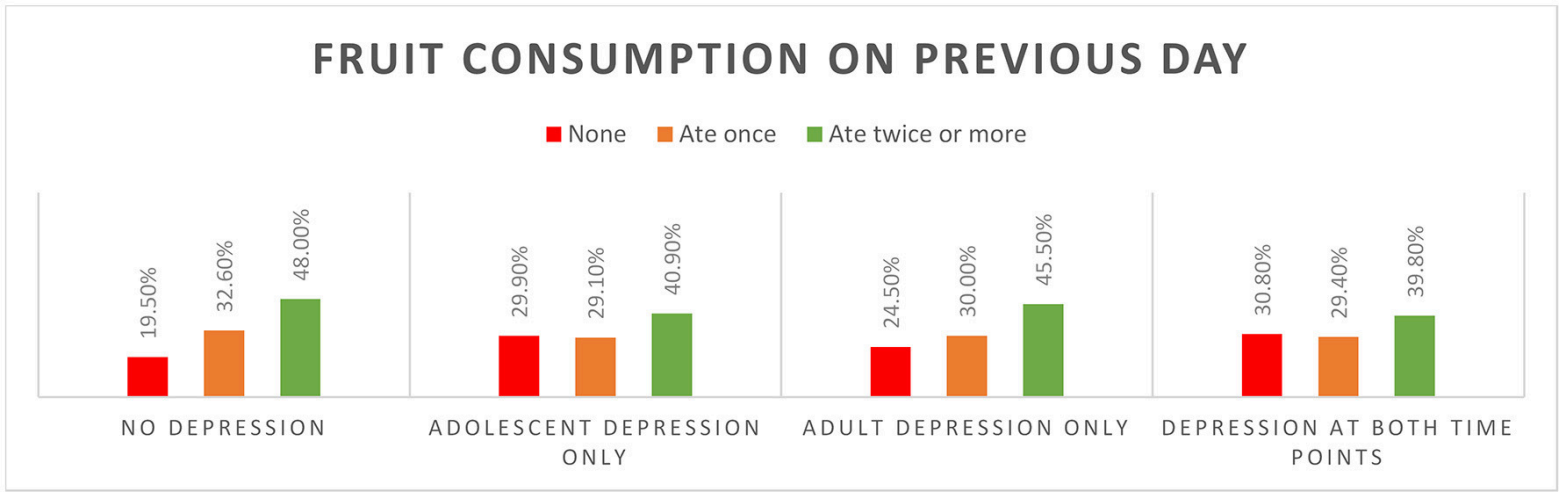
Fruit consumption on previous day by depression status.

**Figure 2:**
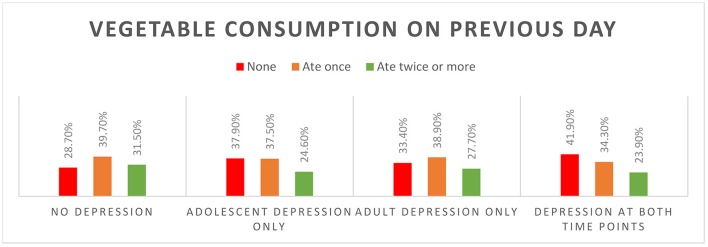
Vegetables consumption on previous day by depression status.

Individuals who never experienced depression were the highest consumers of fruit and vegetables during adolescence (Figure 1; Supplementary Table 1). Most (80%) of the individuals who did not ever experience depression ate fruit on the previous day. The proportion not eating fruit on previous day was higher among those who had depression during adolescence, and among those who went on to experience depression in adulthood. One third of those depressed at both time points did not consume fruit and 42% did not consume any vegetables on the previous day. Vegetable consumption was highest among those who did not experience depression at either time point (Figure 2).

Fruit consumption among males and vegetable consumption among females was prospectively associated with a reduced risk of adult depression in unadjusted models (Table 2). The association between vegetable consumption among females and the risk of adult depression remained significant after controlling for adolescent depression; however, this relationship was attenuated after controlling for age, household income, ethnicity, physical activity and overweight/obesity. Fruit consumption was cross-sectionally related to reduced odds of depression in adolescence in both males and females, both before and after controlling for covariates (Table 3). Vegetable consumption among females was cross-sectionally associated with reduced odds of depression in adolescence.

**Table 2:** Prospective logistic regression models of adult depression (dependent variable) and associations with fruit and vegetable consumption during adolescence (independent variable).

	***Model 1***						***Model 2***						***Model 3***					
**Depress**	**Males**			**Females**			**Males**			**Females**			**Males**			**Females**		
**Adult**	**OR**	**95%CI**	***p***	**OR**	**95%CI**	***p***	**OR**	**95%CI**	***p***	**OR**	**95%CI**	***p***	**OR**	**95%CI**	***p***	**OR**	**95%CI**	***p***
**Fruit**																		
0	Ref.			Ref.			Ref.			Ref.			Ref.			Ref.		
Once	**0.62**	**0.41, 0.94**	**0.025**	0.81	0.57, 1.15	0.239	0.71	0.46, 1.10	0.123	0.93	0.65, 1.35	0.715	0.72	0.46, 1.11	0.139	0.92	0.63, 1.33	0.645
Twice +	**0.64**	**0.44, 0.94**	**0.021**	0.76	0.55, 1.05	0.098	0.73	0.49, 1.09	0.123	0.89	0.63, 1.26	0.526	0.71	0.47, 1.07	0.097	0.88	0.62, 1.26	0.478
**Veg**																		
0	Ref.			Ref.			Ref.			Ref.			Ref.					
Once	0.96	0.65, 1.40	0.815	**0.66**	**0.48, 0.89**	**0.007**	1.00	0.68, 1.48	0.986	**0.72**	**0.53, 1.00**	**0.047**	1.07	0.72, 1.57	0.752	0.74	0.54, 1.02	0.068
Twice +	0.97	0.64, 1.46	0.870	**0.68**	**0.49, 0.94**	**0.019**	1.01	0.66, 1.55	0.951	0.79	0.57, 1.10	0.168	1.02	0.66, 1.56	0.936	0.80	0.57, 1.12	0.201

*Unadjusted (Model 1), adjusted for adolescent depression (Model 2) and further adjusted for age, household income, ethnicity, physical activity, and BMI (Model 3) models are presented*.

*BMI, body mass index; CI, confidence interval; OR, Odds ratio*.

*Bolding indicates significance at p < 0.05*.

**Table 3:** Cross-sectional logistic regression models of adolescent depression (dependent variable) and associations with fruit and vegetable consumption in adolescence (independent variable).

	**Model 1**						**Model 2**					
**Depress**	**Males**			**Females**			**Males**			**Females**		
**Adoles**	**OR**	**95%CI**	***p***	**OR**	**95%CI**	***p***	**OR**	**95%CI**	***p***	**OR**	**95%CI**	***p***
**Fruit**												
None	Ref.			Ref.			Ref.			Ref.		
Once	**0.48**	**0.33, 0.70**	**<0.001**	**0.59**	**0.43, 0.81**	**0.001**	**0.53**	**0.36, 0.77**	**0.001**	**0.68**	**0.49, 0.95**	**0.025**
Twice +	**0.50**	**0.35, 0.71**	**<0.001**	**0.54**	**0.40, 0.73**	**<0.001**	**0.55**	**0.38, 0.80**	**0.002**	**0.62**	**0.45, 0.85**	**0.003**
**Veg**												
None	Ref.			Ref.			Ref.			**Ref**.		
Once	0.79	0.56, 1.11	0.171	**0.65**	**0.49, 0.85**	**0.002**	0.80	0.56, 1.13	0.206	**0.69**	**0.52, 0.93**	**0.014**
Twice +	0.79	0.54, 1.16	0.232	**0.54**	**0.39, 0.73**	**<0.001**	0.87	0.59, 1.28	0.475	**0.64**	**0.46, 0.88**	**0.006**

*Unadjusted (Model 1), and adjusted for age, household income, ethnicity, physical activity, and BMI (Model 2) models are presented. BMI, body mass index; CI, confidence interval; OR, Odds ratio*.

*Bolding indicates significance at p < 0.05*.

## Discussion

Fruit and vegetable consumption during adolescence was predictive of adult depression in univariate models, however, this relationship was attenuated once adjusted for adolescent depression in males and by further adjustment for key confounding variables in females. In cross-sectional analyses, fruit (males and females) and vegetable (females only) consumption was related to adolescent depression, both before and after controlling for covariates. Consumption patterns by depressive status were as expected; fruit and vegetable consumption was highest among individuals who did not experience depression during adolescence or adulthood.

Our findings are somewhat inconsistent with previous literature that suggests that diet during adolescence is predictive of both concurrent and future mental health outcomes (26, 29). It has been suggested that it is fruit and vegetables, as a component of wider healthy diet, that promotes and supports mental and brain health (15, 26, 30). This was highlighted in research by (4) who proposed that it is the combination of foods and habitual dietary patterns over time, that hold implications for inflammation and oxidative stress which are contributing pathways to mood and emotional dysfunction that underpins depression (4). In light of the current results, it is possible that failing to incorporate the wider diet beyond fruit and vegetables, resulted in failure to capture potential predictive potential of future depression. In particular, given the established relationship between unhealthy diets and adolescent (31) and adult (1) depression, failure to assess and model the intake of other food groups, including processed and “junk” foods, may have limited our findings. This suggests that promoting fruit and vegetable consumption alone may not be enough to support prevention efforts. It is also known that mental health and diet relationship is considered bi-directional in that depressed states can impact upon appetite, hedonic capacity and food and dietary decisions (32). Recent evidence demonstrates poorer diet quality at a population level, among those living with mental illness, compared to those who do not experience mental illness (9). This relationship has been shown to be independent of relevant confounders including age, gender, BMI, education, and social deprivation. The cross-sectional findings here during adolescence supports this bi-directionality.

Eating vegetables once a day during adolescence was predictive of reduced odds of depression as an adult among females, compared to females who did not consume vegetables on the previous day. Sex differences have been established both in experiences and trajectory of depressive disorders, and also in health behaviors across the life span (5, 33). For example, our recent study showed that adolescent females were close to twice as likely to meet fruit and vegetable consumption recommendations compared to males (33). In emerging adulthood (18–30 years), females were less likely to be habitual sugar sweetened beverage consumers and more likely to meet physical activity recommendations, compared to emerging adult males. It is possible that young females who are regular consumers of vegetables are also engaging in other protective behaviors that might in combination offset depression. This finding is particularly important with a disproportionate risk of depression increasing with age among adolescent females compared to adolescent males (5). Males and females experience similar level of risk of depression in early adolescence, and by early adulthood females are twice more likely to experience depression than males of the same age (34).

It is possible that the influence of socio-economic circumstances were inadequately captured in this study. For example, whilst household income was included in fully adjusted models, participants' socio-economic circumstances may have changed with their transition from adolescence to adulthood. It is possible that life experiences occurring in early adulthood such as leaving school, living independently, commencing studies or full-time employment, may have influenced adult mental health and the potential contribution of these factors was not adequately captured. Higher rates of depression are consistently seen in neighborhoods experiencing disproportionate socio-economic disadvantage (35). There are environmental challenges in such communities, such as greater density of fast food outlets in place of fresh wholefood stores (36). The influence of socio-economic circumstances on mental and physical health outcomes are well-established, and it is possible that the failure to identify significant relationships in this study was due to inadequate capturing of such circumstances.

The expectation for fruit and vegetable consumption to impact upon current and future mental health is supported by physiological mechanistic pathways. Several plausible mechanisms have been proposed as to why fruit and vegetable consumption may confer protective benefits. Fruits and vegetables are rich in essential micronutrients such as folate, magnesium and selenium. Deficiencies in nutrients, such as folate, have been implicated in the pathogenesis of depression (37). Folate is an essential co-factor in metabolic pathways required for the synthesis of neurotransmitters, such as noradrenaline and serotonin (38), and may further exert protective benefits by decreasing homocysteine levels (39). Further, adequate intake of magnesium may reduce inflammation (40), and protect against chronic, low-grade inflammation which is implicated in depression (11).

Non-nutrient components of fruits and vegetables, such as polyphenols, also strengthen our rationale for fruit and vegetables to protect against depression. Polyphenols refers to a wide class of compounds that as antioxidants, may protect cells against oxidative damage (41) to positively influence brain health (42). Polyphenols may also act as prebiotics within the gut, and along with other dietary fibers, may contribute to the health of the commensal bacteria that reside within the gastrointestinal tract (i.e., the gut microbiota). Numerous studies suggest that the gut microbiota may play a key role in the pathophysiology of depression (43); thus, modulation of the microbiome by prebiotics and dietary fibers may have associated mental health benefits.

## Strengths and limitations

There were study design elements that limited this study. In particular, the measurement of fruit and vegetable consumption was self-reported, and this is likely to have introduced biases to the analysis such as social desirability bias. Moreover, there is large measurement error arising from dietary questionnaires and this would have attenuated the strength of the detected associations (44). On the other hand, single item fruit and vegetable consumption items have been shown to correlate with comprehensive dietary recall measures, and are widely used in population health research (44). In addition, our study examined fruit and vegetable consumption during adolescence only, and our findings could have been further explored with the inclusion of adult dietary behaviors. It is possible that dietary habits changed over time, but we did not have data to assess this. As identified, this study accounted for household income as a proxy for socio-economic status during adolescence. However, it is likely that social and economic changes were likely to have affected health behavioral and depressive outcomes in the transition from adolescence to adulthood. Our study failed to account for the impact of such changes. Our study was strengthened by the use of longitudinal cohort data, which were sampled to be representative of the US population. Despite this, the extent to which findings can translate to current adolescent health is limited. For example, the social environment and daily habitual lifestyle of adolescents is different to that experienced two decades ago and such experiences are likely to impact upon health outcomes adulthood. Lastly, the CES-D is a self-reported measurement of depression and our study was limited in that no clinical depression assessment was available. However, it has been commonly used in epidemiological studies and is widely accepted as an appropriate tool for the measurement of depression (23, 45).

## Conclusion

The findings of this study offer further support for the role and importance of lifestyle behaviors, and in particular diet, for understanding experiences of mental health during adolescence. The wider evidence to date suggests that fruit and vegetable consumption could be an important predictor of future depression, and our null findings may be due to the limitations of our dietary assessment, which failed to capture overall dietary patterns and was likely affected by measurement error. Increasing consumption of fruit and vegetables is a highly valuable public health message in terms of preventing chronic and non-communicable diseases. The potential for prevention of depression to be included in this public health messaging is promising, given the current burden associated with depression and lifestyle-driven diseases. Given that diet and other lifestyle behaviors are largely shaped by social and economic circumstances, we propose that such factors, in addition to comprehensive dietary and other lifestyle behaviors, are incorporated into population-level mental health prevention research. Optimal prevention strategies are likely to be achieved through assuming complex interconnecting relationships between such drivers of health across the lifespan.

## Ethics statement

This study was carried out in accordance with the recommendations of University of North Carolina School of Public Health Institutional Review Board guidelines that are based on the Code of Federal Regulations on the Protection of Human Subjects 45CFR46: http://www.hhs.gov/ohrp/humansubjects/guidance/45cfr46.html Add Health participants provided written informed consent for participation in all aspects of Add Health.

## Author contributions

EH developed the study design, conducted the analyses, and drafted the manuscript. MH contributed to the manuscript and revised for intellectual content. AR and FJ revised the manuscript for intellectual content. All authors read and approved the final draft for submission.

### Conflict of interest statement

The funding bodies had no involvement in any aspect of this study including; study design, analysis and interpretation of data, writing of the manuscript, nor the decision to submit the manuscript for publication. FJ is currently writing two books for commercial publication and has a personal belief that good diet quality is important for mental and brain health. The remaining authors declare that the research was conducted in the absence of any commercial or financial relationships that could be construed as a potential conflict of interest.
