# Supplementary material for: Does Fruit and Vegetable Consumption During Adolescence Predict Adult Depression? A Longitudinal Study of US Adolescents
Source: Front Psychiatry. 2018 Nov 13;9:581. doi: 10.3389/fpsyt.2018.00581 (PMC6243081; doi:10.3389/fpsyt.2018.00581)
Supplement: Supplementary file 1 [file Table_1.DOCX]

**Supplementary Table 1. Fruit and vegetable consumption patterns among individuals (i) never depressed, (ii) depression during adolescence only, (iii) depressed during adulthood only, (iv) depressed at both time points, expressed as N (%).**

| **Adolescent fruit and vegetable consumption on previous day** | **No depression**  **N=2,512 (68.0)** | **Depression during adolescence only N=491 (13.3)** | **Depression during adulthood only N=404 (10.9)** | **Depression at both time points N=289 (7.8)** | **Total N=3,696** |
| --- | --- | --- | --- | --- | --- |
| **Fruit** |  |  |  |  |  |
| Didn’t eat | 489 (19.5) | 147 (29.9) | 99 (24.5) | 89 (30.8) | 824 (22.3) |
| Ate once | 818 (32.6) | 143 (29.1) | 121 (30.0) | 85 (29.4) | 1,167 (31.6) |
| Ate twice or more | 1,205 (48.0) | 201 (40.9) | 184 (45.5) | 115 (39.8) | 1,705 (46.1) |
| **Vegetables** |  |  |  |  |  |
| Didn’t eat | 722 (28.7) | 186 (37.9) | 135 (33.4) | 121 (41.9) | 1,164 (31.5) |
| Ate once | 998 (39.7) | 184 (37.5) | 157 (38.9) | 99 (34.3) | 1,438 (38.9) |
| Ate twice or more | 792 (31.5) | 121 (24.6) | 112 (27.7) | 69 (23.9) | 1,094 (29.6) |

*Proportions may not total 100% due to rounding
